# Novel Use of Generalizability Theory to Optimize Countermovement Jump Data Collection

**DOI:** 10.3390/sports13030085

**Published:** 2025-03-12

**Authors:** Alan Huebner, Jonathon R. Lever, Thomas W. Clark, Timothy J. Suchomel, Casey J. Metoyer, Jonathan D. Hauenstein, John P. Wagle

**Affiliations:** 1 Sports Performance, University of Notre Dame, Notre Dame, IN 46556, USA; alan.huebner.10@nd.edu (A.H.); casey.metoyer@gmail.com (C.J.M.); jwagle@nd.edu (J.P.W.); 2 Department of Applied and Computational Mathematics and Statistics, University of Notre Dame, Notre Dame, IN 46556, USA; tclark23@nd.edu (T.W.C.); hauenstein@nd.edu (J.D.H.); 3 Department of Sports Medicine and Nutrition, University of Pittsburgh, Pittsburgh, PA 15260, USA; tis125@pitt.edu

**Keywords:** CMJ, team sport, testing, monitoring, force plate

## Abstract

This study aimed to evaluate the reliability of countermovement jump (CMJ) performance metrics across five NCAA Division I varsity sports using Generalizability Theory (G-Theory). Three hundred male athletes from football, hockey, baseball, soccer, and lacrosse performed three or more CMJs on dual-force platforms. G-Theory was applied to identify variance components and determine reliability coefficients (Φ) for 14 key metrics. Metrics requiring more than three jumps to achieve Φ 0.80 were deemed unreliable. Metric reliability varied by sport and phase of movement. Metrics associated with the eccentric phase (e.g., Eccentric Duration, Deceleration Rate of Force Development Asymmetry) demonstrated lower reliability, often requiring >3 jumps. Reliable metrics across sports included Phase 1 Concentric Impulse and Scaled Power, requiring three trials or fewer. CMJ reliability is sport- and metric-specific. Practitioners should prioritize reliable metrics and adjust protocols to balance data quality and practicality, particularly when monitoring eccentric characteristics.

## 1. Introduction

Precise measurement of physical capabilities is indispensable for optimizing training protocols and preventing injuries in athletes [1]. Countermovement jumps (CMJs) on dual-force platforms are extensively utilized in sports to assess lower-body force-producing capabilities, a critical attribute for athletes across all sporting disciplines [2,3,4]. Specifically, each test yields numerous metrics that can provide underpinning information about how an athlete achieved their performance through analysis of different jump phases (e.g., unweighting, braking, propulsion, landing, etc.) [5]. However, before reporting performance data, it is important to establish the reliability of each of the chosen metrics to ensure that the data are reproducible during multiple trials. The relative and absolute reliability of CMJ performance metrics are typically evaluated by different forms of intraclass correlation coefficients and coefficients of variation, respectively [6]. However, because reliability statistics may vary based on the number of trials, the athlete’s sport background, and the specific metrics being evaluated [7], sport scientists may consider other methods in which reliability may be determined.

Generalizability Theory (G-Theory), introduced by Cronbach and colleagues (1972), provides a robust framework for assessing the reliability of measurements under various conditions [8]. Unlike classical test theory, which offers a reliability coefficient for each unique context (e.g., intra-rater, inter-rater), G-Theory identifies the variance from multiple sources and aggregates them into a single coefficient, facilitating a more comprehensive understanding of measurement reliability [9,10]. This is particularly advantageous in sport settings, where the reliability of performance metrics can be affected by several factors. While G-Theory originated in the field of psychology, researchers in the exercise and sport science fields have used it to examine the variability of isometric force production [11], and motor performance assessments including overarm throwing [12]. Despite the previous findings, no research has examined the reliability of CMJ performances using G-Theory, and thus, further research is warranted.

Applying G-Theory to CMJ assessments involves scrutinizing the consistency of performance metrics across multiple trials and sports. Identifying the point at which these metrics become reliable may enable practitioners to make informed decisions regarding the number of trials necessary to obtain stable and generalizable data, which is crucial for researchers and coaches who depend on these measurements to customize training programs and guide athlete development [13]. With this in mind, the purpose of this study was to apply G-Theory to evaluate the reliability of CMJ performance metrics across different sports teams.

## 2. Methods

### 2.1. Subjects

Three hundred male athletes (M ± SD; body mass = 95.5 ± 19.5 kg, age = 21.2 ± 1.6) from five varsity athletic teams within the same NCAA Division I program were included in this study. The teams in the study included sports such as American football (152 athletes), ice hockey (20 athletes), baseball (41 athletes), soccer (27 athletes), and lacrosse (60 athletes). The decision to include these five groups was based both on having an adequate sample size and the preference to include a collection of sports with characteristically different athletic movements and playing surfaces. Despite the limited sample size guidance for G-Theory analyses, the sample sizes per sport are comparable to or exceed sample sizes utilized in the existing literature pertaining to the reliability of countermovement jump metrics [7,14]. Approval of this study was provided by the university’s Institutional Review Board (IRB) (25-02-9078).

### 2.2. Procedures

Data were collected as part of routine day-to-day sports performance servicing during training throughout the entire academic year and analyzed retroactively. Participants first completed a general warm-up following the RAMP protocol (raise the body temperature and heart rate; activate muscles; mobilize the joints; potentiate with movements) [15] led by a certified strength and conditioning professional. Dual-force platforms (ForceDecks, VALD, Brisbane Australia) were placed on a stable, flat flooring surface and calibrated per the manufacturer’s instructions. Using proprietary, commercially available software (ForceDecks, VALD, Brisbane, Australia), force–time data were acquired at a sampling rate of 1000 Hz and processed immediately after to produce the discrete variables of interest. Each participant stood on the platforms with feet shoulder-width apart. After a quiet standing of at least one second to ensure an accurate capture of system mass [16], they performed a CMJ under the coaching instruction to jump “as high and as fast as you can”, following a countdown of “3, 2, 1, jump”. CMJ trials were performed with hands on hips, a countermovement to a self-selected depth [17], an immediate transition, and the intent to jump as high as possible in one continuous motion [18]. The participants performed a minimum of three, individual jump trials with approximately five seconds between each trial, controlled for by the practitioner. Following the initial three jumps, force–time curves were visually inspected to ensure the integrity of the data. Trials with any technical issues (e.g., incorrect jump technique, not landing on the plate) were excluded and repeated if necessary.

### 2.3. Data Analyses

The variables of interest were selected based on their prevalence of use within a practical high-performance environment and include countermovement depth (CMd), braking impulse (BI), deceleration rate of force development (DRFD), deceleration RFD asymmetry (DRFDa), eccentric duration (ECCdur), force at zero velocity (F@0v), force at zero velocity asymmetry (F@0va), Phase 1 concentric impulse (P1CI), Phase 2 concentric impulse (P2CI), concentric impulse (CI), jump height (JH), take-off velocity (TOv), scaled power (SP), and modified reactive strength index (RSImod) [4,19,20]. A full table of component definitions is included for reference (Table 1). These variables were chosen because, collectively, they capture the key discrete aspects of the force–time curve, providing a comprehensive representation of force production, impulse, and asymmetry across the phases of movement. Additionally, they are widely utilized in practical athlete monitoring settings, offering valuable insights into fatigue management and training adaptations, making them highly relevant for both research and applied practice in high-performance environments.

## 3. Statistical Analyses

G-Theory does not require the assumption of normality in the distribution of measurement errors, allowing it to accommodate a broader range of data distributions compared to traditional reliability analysis methods. Therefore, no assessment of normality was conducted. G-Theory was then used to identify the individual sources of variation including those between athletes (σ^2^_p_, differences in athletes’ “true scores”), trials (σ^2^_t_, systematic differences in scores due to trial number), and residual variance (σ^2^_pt,e_, differences due either to the interaction between persons and trials or some other unmeasured source of variation). These variance components were used to calculate the index of dependability, Φ, a reliability coefficient between 0 and 1, which communicates the proportion of the total variance caused by differences in the objects of measurement (in this case, persons). For this study, the formula for the Φ coefficient was as follows:

$$\Phi =\frac{\sigma_{p}^{2}}{\sigma_{p}^{2}+\sigma_{t}^{2}+\sigma_{pt,e}^{2}}$$

The process of G-Theory was performed in two parts: the G study and the D study. The G study included estimating the variance components described above using an analysis of variance (ANOVA) model and then calculating the Φ coefficient to determine the reliability in which the athlete’s true score could be generalized from a single observed score [21]. The D study (i.e., “Decision” study) included the calculation of the Φ coefficient to determine if the athlete’s true score could be generalized by averaging them over a given number (x) of observations. Therefore, for each of the five varsity teams in the data, a D study was carried out using fourteen key variables produced by the ForceDecks system to determine the optimal number of jumps for each group. For the purposes of this study, variables that necessitate more than 3 jumps to arrive at an adequate Φ coefficient shall be considered “unreliable” metrics. All data cleaning and analyses were performed within the statistical software environment, R (Version 4.4, R Core Team). More specifically, each D-study result was produced using the dtheory package, a user-created package built upon the publicly available gtheory package in R.

## 4. Results

For this study, a “reliable” metric is defined as one requiring three jumps or less to achieve a reliability coefficient of 0.8 or higher within a given population. While this is subjective, sources such as Koo and Li [22] suggest rule-of-thumb ranges for interpreting reliability, i.e., 0.75–0.90 is “good”; thus, we use a slightly more stringent lower threshold.

The results of the D study for the first athletic team of interest, football, are displayed below in Table 2. The most unreliable metrics for this population were F@0va, which would require averaging over nine jumps to achieve adequate reliability, and ECCdur, which would necessitate eight jumps. If these two metrics were to be excluded altogether from the variables of interest, the optimal number of jumps to produce sufficient reliability coefficients for all remaining variables would be five. Rounding out the list of unreliable metrics for the football data are CMd (five jumps), DRFDa (four jumps), and F@0v (four jumps).

The next D study was performed on the hockey team, the results of which are displayed in Table 3. Unlike the football data, the hockey data only contained one unreliable metric—DRFDa. However, given that this variable would require no less than 10 jumps to arrive at a reliability coefficient of 0.8 or higher, this metric would likely need to be excluded from the list of key metrics for this population. Doing so would cause three jumps to become the optimal number of trials for this population.

The third D study, presented in Table 4, was carried out with the baseball team as the population of interest. Like hockey, the D study for baseball resulted in only one unreliable movement metric, though this time it was BI. This variable would need to be averaged over seven jumps, which would likely be too time-consuming for researchers to put into practice. Therefore, as was recommended in the hockey analysis, the appropriate course of action for the baseball data would be to remove this variable from the key ForceDecks variables for this population. Such a conclusion would again cause the optimal number of jumps for the population to become three.

Far and away, the athletic team with the most reliable metrics was soccer, the D study for which is shown in Table 5. While it did contain one “unreliable” metric (DRFDa), the “unreliability” of this metric is debatable due to its required four jumps and, thus, such a label is dependent on the judgment of the analyst. Nevertheless, taking all variables into account, the researcher would be able to retain all variables of interest and require only four jumps on the ForceDecks system from this population to obtain reliable metric data.

Finally, the fifth and final D study was performed on the data from the lacrosse team, which was one of the most problematic of the populations of interest in terms of metric reliability. The results displayed in Table 6 show a total of five unreliable metrics contained within the data for this athletic team. RSImod and DRFDa were the most unreliable, necessitating six and five jumps, respectively, to obtain adequately reliable data. P2CI, JH, and ECCdur would all be considered unreliable metrics as well, as they all require four jumps to arrive at a sufficient Φ coefficient. It might not be practical to ask athletes from this population to jump on the ForceDecks system six times, since doing so would begin to challenge practitioners’ time constraints. Therefore, assuming RSImod has been removed, the optimal number of jumps for the lacrosse team would be either five or four, depending on whether or not the researchers are compelled to include DRFDa in their list of key variables.

## 5. Discussion

The present study evaluated the reliability of various CMJ metrics across five men’s athletic teams—football, hockey, baseball, soccer, and lacrosse. The primary objectives were to identify metrics that achieved suitable reliability (i.e., coefficient ≥ 0.80) within three jump trials and to determine the optimal number of trials required for reliable data acquisition in each sport. Results revealed considerable variability in metric reliability both within and across sports, underscoring the importance of sport-specific considerations when applying force plate technology in high-performance environments.

When considering the variability metric reliability across sports, no metric in the study was universally unreliable across all five sports. DRFDa emerged as the least reliable, requiring 4, 10, 3, 4, and 5 trials for football, hockey, baseball, soccer, and lacrosse, respectively. The substantial number of trials needed for this metric to achieve adequate reliability suggests that it may not be practical for routine athlete monitoring. Monitoring of DRFDa often plays a key role in lower-limb injury risk mitigation and injury rehabilitation, highlighting neuromuscular imbalances [23]. Interestingly, while DRFDa demonstrated a delayed stabilization of reliability, DRFD itself was among the most reliable metrics. This disparity could be attributed to the inherent variability in asymmetry measures, which are influenced by limb-to-limb differences that fluctuate across trials [24]. In contrast, DRFD as an absolute measure likely benefits from being less sensitive to such inter-limb variability, providing a more consistent representation of braking capacity. Overall, metrics like DRFD, P1CI, and SP, which also demonstrated high reliability across all sports, appear to offer practical and dependable tools for athlete monitoring in high-performance environments, averaging just 1.4 trials to reach the reliability threshold of 0.80. When minimizing load would be beneficial to an athlete or time is constrained, practitioners may employ just two jumps to reliably indicate the athlete’s force production, acceleration, movement efficiency [25], and power in relation to their body mass [26].

A key finding of this study is the relatively lower reliability of metrics associated with the eccentric phase of the CMJ, particularly in certain sports. For example, in football, variables such as F@0v and ECCdur require eight or more repetitions to achieve acceptable reliability. This trend of less reliable eccentric-phase metrics was also evident in other sports; however, hockey, soccer, and baseball largely demonstrated reliable eccentric characteristics. Notably, in these three sports, excluding a single eccentric variable (DRFDa for hockey and soccer, and BI for baseball) reduced the number of required trials to three, meeting practical standards for athlete monitoring. While practitioners may choose to retain these variables, selective use of eccentric metrics is recommended [27], particularly when monitoring fatigue and performance, to balance practical feasibility with the need for reliable data [28].

These findings suggest that the variability in eccentric metrics may be influenced more by the data collection process—such as the stability of the baseline period prior to the jump—than by inherent limitations of the processing algorithms [19]. Accurate recognition of movement onset is critical for the reliable processing of eccentric characteristics, highlighting the importance of ensuring a consistent and stable baseline during testing [3,26]. By reducing the number of required trials, practitioners can allocate time to ensure athletes achieve a stable baseline between attempts, thereby enhancing data quality. Recent recommendations advocate for using a meaningful change in the rate of force development to determine movement onset, rather than relying solely on relative or absolute thresholds from system mass [29]. While this investigation employed the widely used practical threshold of a 20 N absolute difference from baseline [4,19,30], the results suggest that incorporating a rate of force development as a criterion—alongside ensuring a stable baseline—may further improve the reliability of eccentric-phase metrics. These adjustments to collection protocols could enhance the practical utility of force plate testing for athlete monitoring.

The findings of this study emphasize the importance of tailoring performance metric selection and testing protocols to the specific demands of each sport. For metrics with high variability, practitioners must carefully balance the trade-offs between the number of trials and the reliability of the data collected. Metrics with high reliability, such as P1CI and SP, should be prioritized in routine assessments to ensure consistent results while minimizing the burden on athletes [31]. While previous recommendations suggest performing three to five jump trials to obtain reliable measures [1,32], this study supports the feasibility of achieving reliable outcomes with just three trials in most cases. This reduction in required trials offers strength and conditioning coaches an opportunity to maximize their weight room sessions, allowing for additional focus on other performance-enhancing activities.

This study highlights the variability in metric reliability across different sports, reinforcing the need for sport-specific calibration of performance assessments. By prioritizing reliable metrics, practitioners can ensure efficient and dependable evaluations, ultimately enhancing the effectiveness of training and athlete monitoring strategies. These findings provide a foundation for continued refinement of force plate testing protocols, contributing to improved data quality and practical outcomes in sports performance analysis. Future research should extend these findings by examining the reliability of performance metrics across a wider range of sports and athletic populations. Additionally, exploring factors such as technical execution and athlete fatigue could provide deeper insights into the sources of variability and further refine the practical application of force plate technology in high-performance environments.

## Figures and Tables

**Table 1:** Definitions of countermovement jump metrics included in the analysis.

Metric	Definition
Countermovement Depth (cm)	The maximum negative displacement of the center of mass (CoM) during the eccentric phase of the jump.
Braking Impulse (N·s)	The total impulse generated from the peak negative force to the point at which the CoM velocity reaches zero (end of the eccentric phase).
Deceleration Rate of Force Development (N/s)	The rate of force change calculated from the peak negative velocity to the force value at which the CoM velocity reaches zero (end of the eccentric phase).
Deceleration Rate of Force Development Asymmetry (%)	The percentage difference in deceleration RFD between limbs, calculated as the limb difference divided by the limb sum.
Eccentric Duration (s)	The time elapsed from the onset of movement to the point where the CoM velocity reaches zero (end of the eccentric phase).
Force at Zero Velocity (N)	The force exerted at the point where the CoM velocity reaches zero, calculated using the impulse–momentum relationship.
Force at Zero Velocity Asymmetry (%)	The percentage difference in force at zero velocity between limbs, calculated as the limb difference divided by the limb sum.
P1 Concentric Impulse (N·s)	The impulse generated during the first half of the concentric phase (from zero velocity to the midpoint of the concentric phase).
P2 Concentric Impulse (N·s)	The impulse generated during the second half of the concentric phase (from the midpoint of the concentric phase to triple extension).
Concentric Impulse (N·s)	The total impulse generated from the beginning of the concentric phase (force at zero velocity) to take-off (when the system mass achieves zero force).
Jump Height (cm)	The maximum vertical displacement of the CoM during flight, estimated using the impulse–momentum method.
Take-off Velocity (m/s)	The velocity of the CoM at take-off, estimated using the impulse–momentum method.
Scaled Power (W/kg^2/3^)	The power output near take-off, calculated as the product of force and time divided by body mass raised to the two-thirds power (i.e., allometric scaling).
Reactive Strength Index Modified (AU)	Jump height divided by the total jump duration (sum of the eccentric and concentric durations).

**Table 2:** Reliability coefficient by countermovement jump metric from the football D study.

	Jump Trials			
Metric	1	3	5	7
Countermovement Depth	0.46	0.72	0.81	0.86
Braking Impulse	0.60	0.82	0.88	0.91
Eccentric Deceleration RFD	0.85	0.95	0.97	0.98
Eccentric Deceleration RFD Asymmetry	0.52	0.76	0.84	0.88
Eccentric Duration	0.35	0.61	0.73	0.79
Force at Zero Velocity	0.54	0.78	0.85	0.89
Force at Zero Velocity Asymmetry	0.31	0.58	0.70	0.76
P1 Concentric Impulse	0.80	0.92	0.95	0.97
P2 Concentric Impulse	0.64	0.84	0.90	0.92
Concentric Impulse	0.76	0.90	0.94	0.96
Takeoff Velocity	0.90	0.97	0.98	0.99
Scaled Power	0.88	0.96	0.97	0.98
Jump Height Impulse Momentum	0.75	0.90	0.94	0.96
RSI-modified Impulse Momentum	0.98	0.99	1.00	1.00

**Table 3:** Reliability coefficient by countermovement jump metric from the ice hockey D study.

	Jump Trials			
Metric	1	3	5	7
Countermovement Depth	0.83	0.94	0.96	0.97
Braking Impulse	0.81	0.93	0.96	0.97
Eccentric Deceleration RFD	0.70	0.87	0.92	0.94
Eccentric Deceleration RFD Asymmetry	0.30	0.56	0.68	0.75
Eccentric Duration	0.61	0.82	0.89	0.92
Force at Zero Velocity	0.84	0.94	0.96	0.97
Force at Zero Velocity Asymmetry	0.77	0.91	0.94	0.96
P1 Concentric Impulse	0.95	0.98	0.99	0.99
P2 Concentric Impulse	0.93	0.98	0.99	0.99
Concentric Impulse	0.99	1.00	1.00	1.00
Takeoff Velocity	0.93	0.98	0.99	0.99
Scaled Power	0.94	0.98	0.99	0.99
Jump Height Impulse Momentum	0.93	0.98	0.99	0.99
RSI-modified Impulse Momentum	0.78	0.91	0.95	0.96

**Table 4:** Reliability coefficient by countermovement jump metric from the baseball D study.

	Jump Trials			
Metric	1	3	5	7
Countermovement Depth	0.87	0.95	0.97	0.98
Braking Impulse	0.40	0.67	0.77	0.82
Eccentric Deceleration RFD	0.85	0.94	0.97	0.98
Eccentric Deceleration RFD Asymmetry	0.63	0.84	0.90	0.92
Eccentric Duration	0.86	0.95	0.97	0.98
Force at Zero Velocity	0.89	0.96	0.98	0.98
Force at Zero Velocity Asymmetry	0.80	0.92	0.95	0.97
P1 Concentric Impulse	0.92	0.97	0.98	0.99
P2 Concentric Impulse	0.85	0.95	0.97	0.98
Concentric Impulse	0.92	0.97	0.98	0.99
Takeoff Velocity	0.88	0.96	0.97	0.98
Scaled Power	0.95	0.98	0.99	0.99
Jump Height Impulse Momentum	0.89	0.96	0.98	0.98
RSI-modified Impulse Momentum	0.91	0.97	0.98	0.99

**Table 5:** Reliability coefficient by countermovement jump metric from the soccer D study.

	Jump Trials			
Metric	1	3	5	7
Countermovement Depth	0.81	0.93	0.96	0.97
Braking Impulse	0.71	0.88	0.92	0.95
Eccentric Deceleration RFD	0.77	0.91	0.94	0.96
Eccentric Deceleration RFD Asymmetry	0.54	0.78	0.86	0.89
Eccentric Duration	0.69	0.87	0.92	0.94
Force at Zero Velocity	0.91	0.97	0.98	0.99
Force at Zero Velocity Asymmetry	0.82	0.93	0.96	0.97
P1 Concentric Impulse	0.95	0.98	0.99	0.99
P2 Concentric Impulse	0.87	0.95	0.97	0.98
Concentric Impulse	0.98	0.99	1.00	1.00
Takeoff Velocity	0.92	0.97	0.98	0.99
Scaled Power	0.93	0.98	0.99	0.99
Jump Height Impulse Momentum	0.93	0.98	0.99	0.99
RSI-modified Impulse Momentum	0.83	0.94	0.96	0.97

**Table 6:** Reliability coefficient by countermovement jump metric from the lacrosse D study.

	Jump Trials			
Metric	1	3	5	7
Countermovement Depth	0.76	0.90	0.94	0.96
Braking Impulse	0.72	0.89	0.93	0.95
Eccentric Deceleration RFD	0.83	0.94	0.96	0.97
Eccentric Deceleration RFD Asymmetry	0.47	0.73	0.82	0.86
Eccentric Duration	0.52	0.77	0.85	0.89
Force at Zero Velocity	0.78	0.91	0.95	0.96
Force at Zero Velocity Asymmetry	0.77	0.91	0.94	0.96
P1 Concentric Impulse	0.73	0.89	0.93	0.95
P2 Concentric Impulse	0.52	0.77	0.85	0.89
Concentric Impulse	0.64	0.84	0.90	0.93
Takeoff Velocity	0.86	0.95	0.97	0.98
Scaled Power	0.57	0.80	0.87	0.90
Jump Height Impulse Momentum	0.56	0.79	0.87	0.90
RSI-modified Impulse Momentum	0.42	0.68	0.78	0.83

## Data Availability

Data may be made available upon reasonable request and approval from the institution.
